# Transforming Agricultural and Sulfur Waste into Fertilizer: Assessing the Short-Term Effects on Microbial Biodiversity via a Metagenomic Approach

**DOI:** 10.3390/life14121633

**Published:** 2024-12-09

**Authors:** Angela Maffia, Riccardo Scotti, Thomas Wood, Adele Muscolo, Alessandra Lepore, Elisabetta Acocella, Giuseppe Celano

**Affiliations:** 1 Department of AGRARIA, ‘Mediterranea’ University of Reggio Calabria, Feo di Vito, 89122 Reggio Calabria, Italy; angela.maffia@unirc.it; 2 NIAB, Cambridge Pathology, 93 Lawrence Weaver Road, Cambridge CB3 0LE, UK; riccardo.scotti@crea.gov.it (R.S.); tom.wood@niab.com (T.W.); 3 Consiglio per la Ricerca in Agricoltura e l’Analisi dell’Economia Agraria (CREA), Research Centre for Vegetable and Ornamental Crops, Via Cavalleggeri 51, 84098 Pontecagnano Faiano, Italy; 4 Department of Pharmacy, University of Salerno, Via Giovanni Paolo II, 84084 Fisciano, Italy; alepore@unisa.it (A.L.); e.acocella@studenti.unisa.it (E.A.); gcelano@unisa.it (G.C.)

**Keywords:** organic waste, sulfur, olive pomace, metagenomics, biodiversity, microbiome, *Corylus avellana*

## Abstract

Fungi and soil bacteria are vital for organic matter decomposition and biogeochemical cycles, but excessive synthetic fertilizer use contributes to soil degradation and loss of biodiversity. Despite this, about 97% of soil microorganisms are unculturable, making them difficult to study. Metagenomics offers a solution, enabling the direct extraction of DNA from soil to uncover microbial diversity and functions. This study utilized metagenomics to analyze the rhizosphere of two-year-old *Tonda di Giffoni* hazelnut saplings treated with synthetic NPK, composted olive pomace, and an innovative fertilizer derived from sulfur-based agro-industrial waste stabilized with bentonite clay. Using 16S rDNA for bacteria and ITS2 for fungi, Illumina sequencing provided insights into microbial responses to different fertilizer treatments. The results highlighted a significant increase in the abundance of beneficial microorganisms such as Thiobacillus, Pseudoxanthomonas, and Thermomyces, especially when organic materials were included. Additionally, microbial biodiversity improved with organic inputs, as shown by increased species richness (Chao1) and diversity (Bray-Curtis) greater than 20% compared with NPK and unfertilized soils (CTR). These findings emphasize the importance of organic fertilization in enhancing soil microbial health, offering a sustainable approach to improving soil quality and hazelnut productivity.

## 1. Introduction

Hazelnut (*Corylus avellana* L.), a member of the Betulaceae family, ranks as the second most widely grown nut in the world, following only almonds [1]. The main hub of hazelnut production is located along the Black Sea coast of Turkey, Romania, Moldova, and Bulgaria, with other significant cultivation in Italy, the United States, and Spain [2]. To meet the growing consumer demand for hazelnuts and increase productivity, it is imperative to adopt strategies that ensure robust yields while preserving soil and the environment. Several studies have been conducted to improve the productivity and fertility of hazelnut cultivation [3,4,5] but as reported by Vincze et al. [6], a better understanding of the role of microbes in agroecosystem functioning in the context of plant growth and soil fertility is critical for sustainable agricultural production. Plant nutrition is closely influenced by the microbial community in the rhizosphere [7]. The soil rhizosphere is a micro-ecosystem in which the composition and structure of microorganisms can influence nutrient transformations in the soil, nutrient uptake by plants, and, thus, plant growth and development [8]. According to Roeland et al. [9], the rhizosphere functions as a unique region where the interaction between the plant, root, and soil microbiome takes place; in fact, as stated by Philippot et al. [10], the rhizosphere is one of the most dynamic interfaces on Earth.

In recent years, in response to climate change and the widespread use of synthetic/mineral fertilizers, the effects of different fertilization techniques on the rhizosphere microbial community have begun to be studied [7,11,12].

A study by Chavez-Romero et al. [13] states that the application of organic fertilizers has beneficial effects compared to inorganic fertilizers in promoting the diversity of soil microbial communities and enriching the soil with organic carbon, nitrogen, and other nutrients. Dai X.B et al. [14] observed that the increased application of synthetic nitrogen and phosphate fertilizers changed the composition of the soil microbial community by reducing the abundance of gram-positive bacteria such as actinomycetes. Guo et al. [15] found that synthetic fertilization negatively influenced microbial communities in the rhizosphere, and that positive effects on soil microbial communities were observed with the addition of organic fertilizer compared to chemical fertilization [16]. Similarly, Legrand et al. [17] found greater bacterial richness and uniformity with manure application compared to chemical fertilization. In contrast, Orr et al. [18] observed no discernible influence on the microbial community when comparing conventional and organic farming systems. The composition and biodiversity of the observed microbiota were attributed exclusively to environmental and soil chemical variables.

Although several previous studies have focused mainly on long-term field experiments and organic fertilizers can have an impact even in the short term, within a single growing season, especially on crop productivity [19,20]. Nevertheless, no study has yet been conducted on the evaluation of mineral and organic synthetic fertilizers on hazelnut saplings in the short term, specifically on the influence of these fertilizations on rhizosphere microbial processes. Studies that have employed culture-based methods [21,22] capture only a limited fraction of the overall microbial diversity. In contrast, next-generation sequencing (NGS) techniques offer a powerful approach to achieving a more comprehensive understanding of microbial community composition and diversity [23].

The aim of this study is to evaluate the short-term changes in microbial diversity and community in the rhizosphere of hazelnut saplings. The rhizosphere microbial community of unfertilized hazelnut saplings was compared with the rhizosphere microbial community of hazelnut saplings fertilized with synthetic commercial NPK fertilizer (NPK), composted olive pomace (OP), sulfur bentonite (SB), and sulfur bentonite + composted olive pomace (SBOP).

SB and SBOP are new fertilizers obtained from agro-industrial wastes composed as follows: SB comprises elemental sulfur (S) residue from the hydrocarbon refining process stabilized with bentonite clay, without composted olive pomace; SBOP comprises elemental S residue from the hydrocarbon refining process stabilized with bentonite clay, with the addition of composted olive pomace. The effectiveness of sulfur-based fertilizers, both with and without an organic component, has been demonstrated in different studies [24,25], but no studies up to now have been carried out on tree species. This study aims to verify if fertilization yields different outcomes compared to the previously studied horticultural plants. The novelty of this study lies in its integration of advanced metagenomic techniques with the evaluation of innovative organic fertilizers to assess their impact on soil microbial communities. By employing 16S rDNA and ITS2 sequencing to profile bacterial and fungal communities, the study provides a detailed view of microbial diversity and functions in the rhizosphere, especially in response to different fertilizers. The application of advanced metagenomic techniques to investigate the effects of different fertilizations on the soil microbiome addresses a critical challenge in soil microbiology: the inability to culture approximately 97% of bacterial species due to limited knowledge of their specific cultivation requirements, including nutritional needs, the physicochemical conditions of their natural environment, and the complex symbiotic or parasitic relationships within microbial communities [26]. The combination of innovative fertilizer evaluation, cutting-edge metagenomic analysis, and a focus on sustainable practices provides a fresh perspective on managing soil health in hazelnut agriculture and contributes to broader efforts in sustainable farming practices.

## 2. Materials and Methods

### 2.1. Fertilizers Manufacturing

The olive pomace compost used in this study was produced at the farm composting plant of the Nuovo Cilento agricultural company, in San Mauro Cilento (SA), Italy.

The chemical characteristics of olive pomace compost are reported in Table 1 and analyzed according to Muscolo et al. [24]. The compost highlights an alkaline pH, moderate electrical conductivity, good organic matter content, and the presence of essential nutrients (anions and cations).

The synthetic fertilizers were produced by the Steel Belt System s.r.l and the process of manufacturing is reported in Panuccio et al. [27]. The composition of different fertilizers is reported in Table 2.

### 2.2. Experimental Design

In February 2023, two-year-old hazelnut saplings var. *Tonda di Giffoni* were placed in 30-L capacity pots filled with sandy loam soil (64% sand, 28% loam, and 8% clay) according to the Agricultural Organization of the United Nations (FAO) [28]. The soil contained an organic matter content of 20 g × kg^−1^, a total nitrogen concentration of 1 g × kg^−1^, and a carbon-to-nitrogen ratio of 12.

Four replications were made per treatment for a total of 20 pots, with the following doses of each treatment per pot:

- 45 g of composted olive pomace (OP).
- 5 g of sulfur bentonite + olive pomace (SBOP).
- 5 g of sulfur bentonite (SB).
- 4.2 g of synthetic fertilizer (NPK).
- Unfertilized soil (CTR).

**Figure 1:**
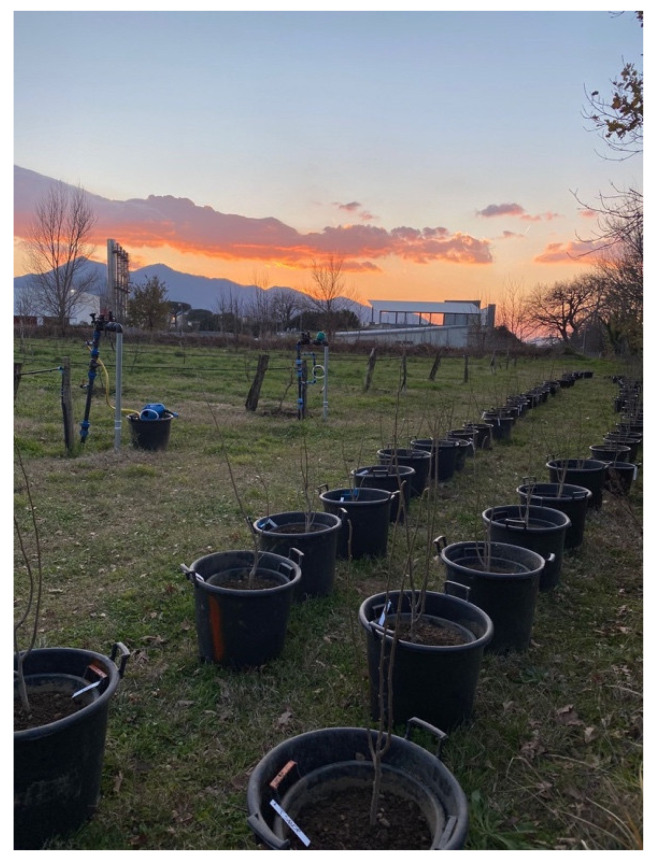
Experimental site and pots with *Corylus avellana* plants and different treatments.

For each treatment, fertilizer was applied twice before flowering in February and before vegetative growth in June. To limit weed development and avoid excessive soil overheating, the pots were isolated by placing them in larger-diameter containers (50 cm), and the surface soil and defined chamber were filled with expanded clay (Figure 1). The soil water content, through irrigation with potable water, was always maintained at 70% of field capacity.

### 2.3. Sample Collection and DNA Extraction

**Figure 2:**
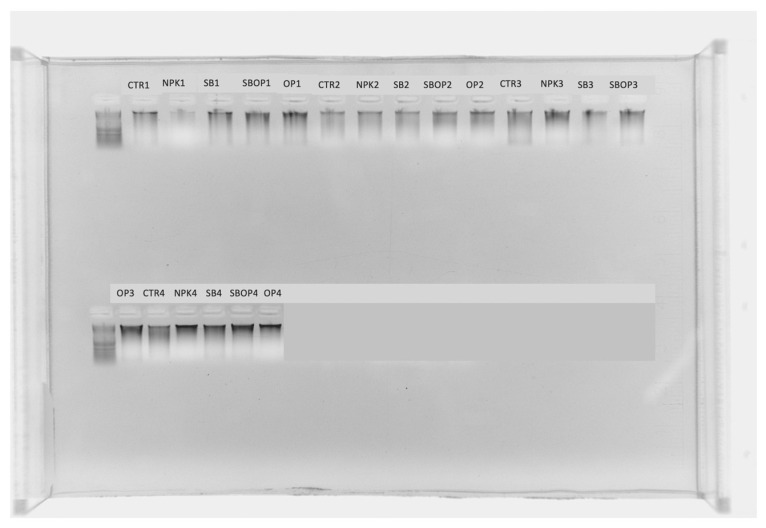
DNA quality control of DNA extraction samples obtained using a Luminescence Image Analyzer System (LAS).

In July, rhizosphere sampling was performed using the protocol of Simmon et al. [29], with some modifications: 2 mm of soil attached to the roots was left and a representative section was cut and placed in a 50 mL tube with 25 mL of phosphoric buffer, centrifuged for 10 min at 4000× *g* at 4 °C, and the root was removed to obtain the rhizosphere fraction. Rhizosphere DNA was extracted using a commercial kit DNeasy PowerSoil Pro kit, (Qiagen, Hilden, Germany), following the protocol with one modification: 10 mg of skim milk was added to all the samples, as suggested by Hoshino et al. [30], to increase the DNA extraction yield from volcanic soil. The final DNA concentrations were determined using a NanoDrop 2000 UV-vis spectrophotometer (Thermo Scientific, Wilmington, NC, USA), and the DNA quality was checked using 1% agarose gel electrophoresis on a Luminescence Image Analyzer System (LAS 4000; ImageQuant, GE Healthcare Life Sciences, Little Chalfont, UK) (Figure 2).

DNA samples were screened for the V3–V4 hypervariable regions via PCR using primers 341F (5′-GCGGTAATTCCAGCTCCAA-3′) and 806R (5′-GGACTACNNGGGTATCTAAT) for 16S rDNA. For ITS2, the primers ITS3-2024F (5′-GCATCGATGAAGAACGCAGC-3′) and ITS4-2409R (5′-TCCTCCGCTTATTGATATGC-3′) were used [31]. All PCR reactions were carried out with 15 μL of Phusion^®^ High-Fidelity PCR Master Mix (New England Biolabs, Ipswich, MA, USA); 2 μM of forward and reverse primers, and about 10 ng of template DNA. Thermal cycling consisted of initial denaturation at 98 °C for 1 min, followed by 30 cycles of denaturation at 98 °C for 10 s, annealing at 50 °C for 30 s, and elongation at 72 °C for 30 s. Sequencing libraries were generated using the TruSeq^®^ DNA PCR-Free Sample Preparation Kit (Illumina, San Diego, CA, USA) following the manufacturer’s recommendations, and index codes were added. The library quality was assessed on the Qubit@ 2.0 Fluorometer (Thermo Scientific) and Agilent Bioanalyzer 2100 system. Lastly, the library was sequenced on an Illumina NovaSeq 6000 platform (Illumina, San Diego, CA, USA) and 250 bp paired-end reads were generated. The DNA library preparation and sequencing were performed by Novogene Co, Ltd. (Beijing, China, http://www.novogene.com/ accessed on 1 May 2024). The raw sequence files generated (fastq files) underwent quality control analysis with FastQC.

### 2.4. Data Analysis

The NGS datasets were analyzed using the EBI Metagenomics service pipeline, which provides quality control, taxonomic analysis based on SSU rDNA sequences, and sequence assembly (MGnify. 2023. “Analysis Pipeline V5.0” 9 November 2023. https://docs.mgnify.org/src/docs/analysis.html accessed on 9 November 2023).

### 2.5. Biodiversity Assessment and Statistical Analysis

Sequence data from the rhizosphere soil community was analyzed using Microbiome Analyst 2.0 (https://www.microbiomeanalyst.ca/) [32]. Microbial biodiversity was assessed to quantify differences between groups at two levels: within-samples (alpha-diversity) and between samples (beta-diversity) [33]. From the different six measures supported in Microbiome Analyst described by Chong et al. [34], the Chao1 index [35] and Shannon index [36,37] were selected. From the five different beta-diversity indexes supported by Microbiome Analyst, “Bray–Curtis dissimilarity” [38] was utilized. The index measures the compositional dissimilarly between the microbial communities based on the counts of each sample. Microbiome Analyst can measure Beta-diversity using PCoA or nonmetric multidimensional scaling (NMDS); for this study, PCoA was selected because it maximizes the linear correlation between samples [34]. For identifying microbial taxa that were significantly different between groups, LEfSe (Linear discriminant analysis Effect Size), a non-parametric statistical method, was selected. A significance level of *p* < 0.05 and LogLDA score of ±2 was applied. The jveen tool (INRAE, Toulouse, France) [39] was used to compare the genera of fungi and bacteria that were found to be statistically significant using LeFSE. ‘Class’ level was selected for bacteria and fungi for generating the Heatmap outputs. The distance between data points in the clustering input is measured with the standard Euclidean (as-the-crow-files) distances. All sequences were deposited at the European Nucleotide Archive (ENA, http://www.ebi.ac.uk/ena accessed on 15 January 2024) under project number PRJEB70816 for bacteria and PRJEB68325 for fungi.

## 3. Results

Sequencing generated a total of 1656509 (16S rDNA) and 1749133 (ITS2) sequences, respectively, for 20 samples, classified into 1202 OTUs for the 16S rDNA and 1642 OTUs for the ITS2.

### 3.1. Bacteria Taxa Abundance

**Figure 3:**
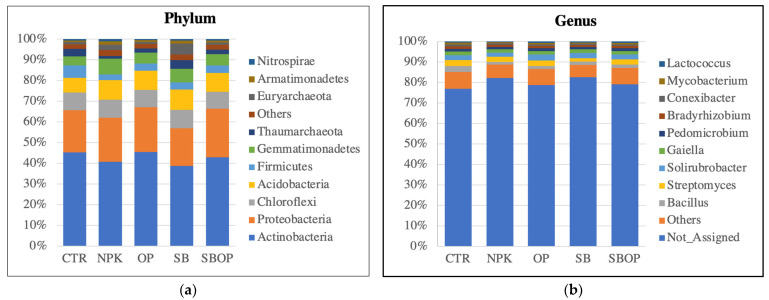
The top 10 bacteria taxa relative abundance for phylum (**a**) and genus (**b**) of soils treated with synthetic fertilizer (NPK), olive pomace (OP), sulfur bentonite (SB), and sulfur bentonite + olive pomace (SBOP). CTR is unfertilized soil.

Results showed that amongst the 10 most abundant bacterial phyla, *Actinobacteria* were the most dominant, constituting 46% in rhizosphere soil treated with OP, 45% in control soil, 43% in SBOP-treated soil, 41% in soil with NPK, and 39% in SB-treated soil (Figure 3a).

The second most abundant phylum was the *Proteobacteria*: representing 23% in SBOP-treated soil, 22% in soil with OP, 21% with NPK, 20% in CTR soil, and 18% in SB-treated soil. *Acidobacteria* increased in all treated soils, compared to CTR (7%), especially with SB (10%).

About 76% of the genus classification was not identified. The genus *Bacillus* was most prevalent in CTR control plants (3%) but decreased with SB (2%) and SBOP (2%) and accounted for only 1% in NPK and OP treatments. Contrastingly, the genus *Streptomyces* was present at an equal percentage of 3% in all treatments except for SB-treated plants where it decreased to 2%. (Figure 3b).

### 3.2. Fungi Taxa Abundance

**Figure 4:**
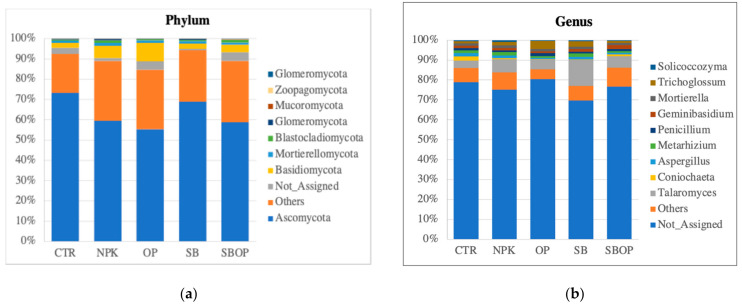
The top 10 fungi taxa abundance for phylum (**a**) and genus (**b**).

Taxonomic classification showed that the most abundant phylum among the samples was *Ascomycota*. The unfertilized CTR soils had the highest percentage of *Ascomycota* fungi (73%), followed by SB treatment (69%), SBOP (59%), NPK (59%), and OP (55%). In addition, an abundance of organisms classified as others, because they do not belong to the kingdom of fungi, was detected, mainly belonging to the phylum *Streptophyta*. Fungi belonging to the phylum *Streptophyta* showed an increase compared to the control (19%) across all treatments: SB (25%), NPK (29%), OP (29%), and SBOP (30%). Fungi from the phylum *Basidiomycota* were predominantly found in the OP treatment (9%) (Figure 4a).

About 76% of the genus classification was not identified. The most abundant genus for all samples was *Talaromycetes*, especially in those treated with SB (13%). This relative abundance decreased in soils fertilized with NPK (7%), SBOP (6%), OP (5%) and in control soil (4%) (Figure 4b).

### 3.3. Bacteria Alpha and Beta Diversity

**Figure 5:**
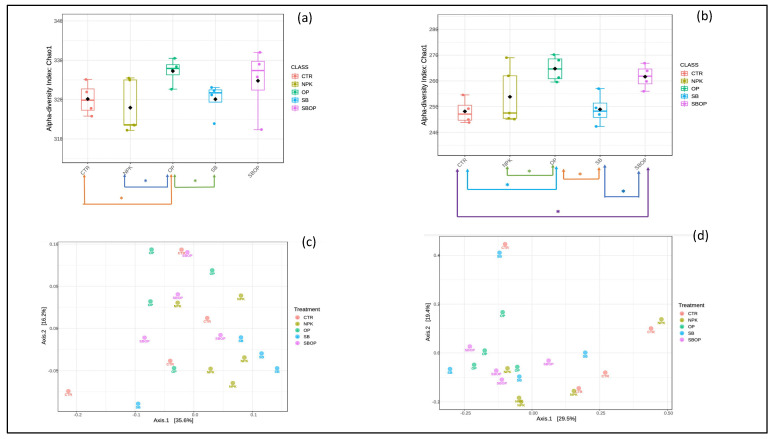
Chao1 index of bacteria (**a**) and fungi (**b**). Statistically significant differences are expressed with (*) for a *p* value < 0.05 through post-hoc pairwise comparison (multiple groups only). Principal Coordinate analysis (PCoA) of bacteria (**c**) and fungi (**d**) calculated with the Bray–Curtis dissimilarity of soils treated with synthetic fertilizer (NPK), olive pomace (OP), sulfur bentonite (SB), and sulfur bentonite + olive pomace (SBOP). CTR is unfertilized soil.

Species richness calculated using the Chao1 index for bacteria OTUs showed that all treatments resulted in an increase compared to control plants (without fertilization) (Figure 5a). Specifically, the largest increase was observed in plants treated with sulfur bentonite + olive pomace fertilizer (SBOP), followed by those treated with olive pomace (OP). Treatment with the synthetic fertilizer (NPK) also resulted in a statistically significant increase, while a smaller increase was noted for sulfur bentonite (SB) treatment (Figure 5a). The results of the post-hoc pairwise comparison (multiple group only) revealed statistically significant differences (*p* < 0.05) between several treatments. Specifically, there was a significant difference between OP and NPK (*p* = 0.034), OP and SB (*p* = 0.039), and CTR and OP (*p* = 0.039). The differences in community composition between samples calculated with the Bray–Curtis index showed a dissimilarity of 35.6% for axis one and a dissimilarity of 16.2% for axis two. (Figure 5c). Although no clear clustering pattern was shown using unsupervised PCoA on Bray–Curtis dissimilarity, the principal coordinate two highlights a trend along the sample distribution, mainly driven by the use of compost on one side and mineral fertilization and sulfur use on the other side. From the result of pairwise PERMANOVA analysis and the multi-testing adjustment, based on the Benjamini–Hochberg procedure (FDR), a statistically significant difference for a *p* value < 0.05 can be seen between the CTR vs. NPK treatments with a *p* value of 0.037, and OP vs. NPK with a *p* value of 0.046 (Supplementary Material Table S1).

### 3.4. Fungi Alpha and Beta Diversity

Species richness, calculated using the Chao1 index for fungal OTUs, demonstrated an increase in all treatments compared to control plants (without fertilization). Specifically, the olive pomace (OP) treatment exhibited the greatest increase in this index, followed by sulfur bentonite + olive pomace (SBOP), and then the synthetic fertilizer (NPK) (Figure 5b).

From the results of the post-hoc pairwise comparison (multiple group only), statistically significant differences (*p* < 0.05) were observed between several treatments. These included OP vs. CTR (*p* = 0.0033), SBOP vs. CTR (*p* = 0.0073), OP vs. SB (*p* = 0.0079), and SBOP vs. SB (*p* = 0.018).

The dissimilarity in community composition between samples calculated using the Bray–Curtis dissimilarly showed a dissimilarity of 29.5% for axis one and a dissimilaritly of 19.4 for axis two (Figure 5c). Although no clear clustering pattern is shown using unsupervised PCoA on the Bray–Curtis index, the second principal coordinate two highlights a trend along the sample distribution mainly driven by the use of compost on one side and mineral fertilization and sulfur use on the other side. From the result of pairwise PERMANOVA analysis and the multi-testing adjustment based on the Benjamini–Hochberg procedure (FDR), a statistically significant difference for a *p* value < 0.05 can be seen between treatments of OP vs. NPK with a *p* value of 0.032, and OP vs. CTR with a *p* value of 0.047 (Supplementary Materials Table S2).

### 3.5. Comparison Analysis with LEfSe, a Heatmap of Relative Abundance, and a Veen Diagram

**Figure 6:**
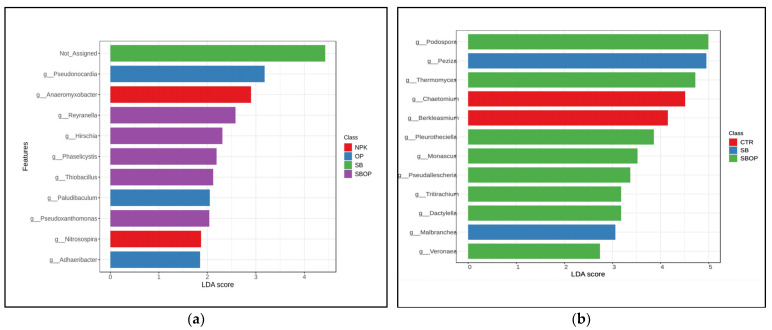
LEfSe results on soil bacterial (**a**) and fungal (**b**) communities. Histogram of the LDA scores computed for features differentially abundant on different treatments. LEfSe scores can be interpreted as the degree of consistent difference in relative abundance between features of analyzed bacterial and fungal communities. The histogram, thus, identifies which clades among all those detected as statistically and biologically differential explain the greatest differences between genus communities.

For bacteria, the linear discriminant analysis effect size (LEfSe) to feature level with the *p*-value cut-off of 0.05 and LogLDA score of 2.0 identified a total of 44 significant features (Supplementary Materials Table S3). There were 10 significant genera observed (Figure 6a).

**Figure 7:**
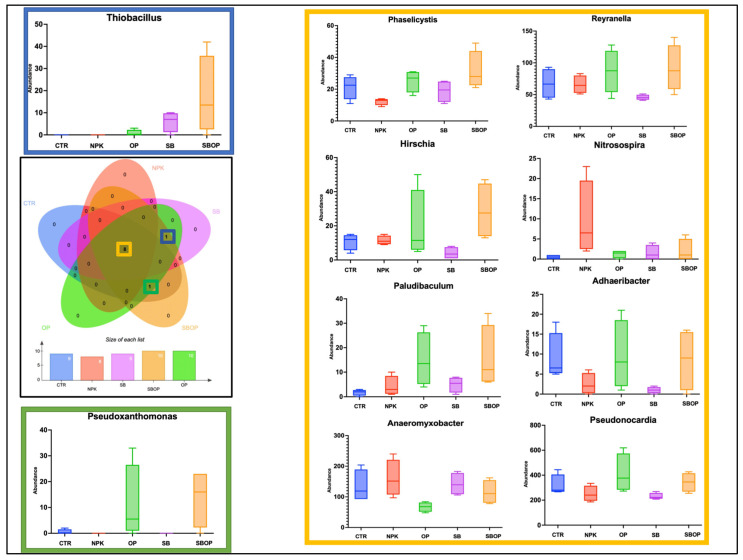
Veen diagram of the genus of bacteria which were found to be statistically significant from linear discriminant analysis effect size (LEfSE) and related box plots.

From the Venn diagram analysis, bacteria belonging to *Pseudonocardia, Phaselicystis, Hirschia, Paludibaculum, Nitrosospira, Anaeromyxobacter, Adhaeribacter*, and *Reyranella* were found to be common across all treatments and the unfertilized soil (CTR) (Figure 7). The genus *Thiobacillus* was common to treatments with OP, SB, and SBOP. In contrast, the genus *Pseudoxanthomonas* was found to be common between treatments with OP and SBOP, as well as in the unfertilized soil (CTR) (Figure 7).

A histogram of the LDA scores was computed for features that were differentially abundant on different treatments. The LEfSe scores can be interpreted as the degree of consistent difference in relative abundance between features of analyzed bacterial and fungal communities (Figure 6). The histogram, thus, identifies which clades among all those detected as statistically and biologically differential explain the greatest differences between genus communities.

The genus *Pseudonocardia* appeared more abundant in the OP treatment, with no significant differences observed compared to other treatments. Meanwhile, the genus *Thiobacillus* was notably absent in both control soil and NPK-fertilized plants but showed an increase with all other treatments, particularly with SBOP. The *Hirschia, Paludibaculum*, and *Reyranella* genera increased in abundance when organic components were added to fertilizers, notably in SBOP and OP treatments. Conversely, the genus *Pseudoxanthomonas* showed an increase compared to the control in OP and SBOP treatments, while decreasing in plants treated with NPK and SB. *Nitrosospira* bacteria were more abundant in NPK-treated soil. However, bacteria belonging to the genus *Anaeromyxobacter* decreased in OP and SBOP treatments. *Adhaeribacter* bacteria decreased across all treatments, with a significant reduction observed in mineral treatments with SB and NPK (Figure 7).

For Fungi, the linear discriminant analysis effect size (LEfSe) was employed up to the taxonomy level feature level, with a *p*-value cutoff of 0.05 and a LogLDA score of 2.0, resulting in a total of 28 significant features (Supplementary Materials Table S4). Among these, 12 significant genera were identified (Figure 6b).

**Figure 8:**
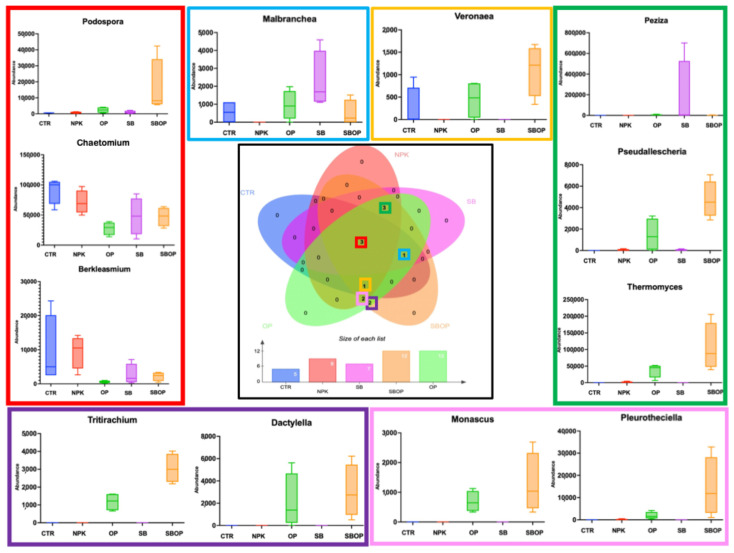
Veen diagram of the genus of fungi which were found to be statistically significant from linear discriminant analysis effect size (LEfSE) and related box plots.

From the Venn diagram analysis (Figure 8), it was observed that the fungi genera *Podospora*, *Chaetomium*, and *Berkleasmium* are common across all treatments and the unfertilized soil (CTR). The genera *Thermomyces, Pseudoallescheria*, and *Peziza* were common across all soils treated with fertilizers. The genus *Malbranchea* was common to the unfertilized soil (CTR), OP, SB, and SBOP treatments but not in NPK treatment. On the other hand, the genus *Veronae* was common to the unfertilized soil (CTR) and all other treatments except SB. Soil treatments with NPK, OP, and SBOP shared the genera *Monascus* and *Pleuroteciella*. Only treatments with OP and SBOP shared the genera *Tritirachium* and *Dactylella*. Fungi of the genus *Pseudoallescheria*, *Thermomyces*, and *Podospora* were predominantly in soil with organic components, such as OP and SBOP fertilizers.

Conversely, fungi of the genus *Chaetomium* and *Berkleasmium* behaved consistently across the OP, SB, and SBOP fertilizer inputs, decreasing compared to soil without fertilizer (CTR) and NPK synthetic fertilizer.

Meanwhile, fungi belonging to the genus *Malbranchea* and *Peziza* showed a significant increase in soil fertilized with SB compared to all other treatments. In contrast, fungi of the genus *Veronae* decreased in treatments with OP and SB compared to unfertilized soil (CTR) and NPK-fertilized soil but increased in soil fertilized with SBOP (Figure 8).

**Figure 9:**
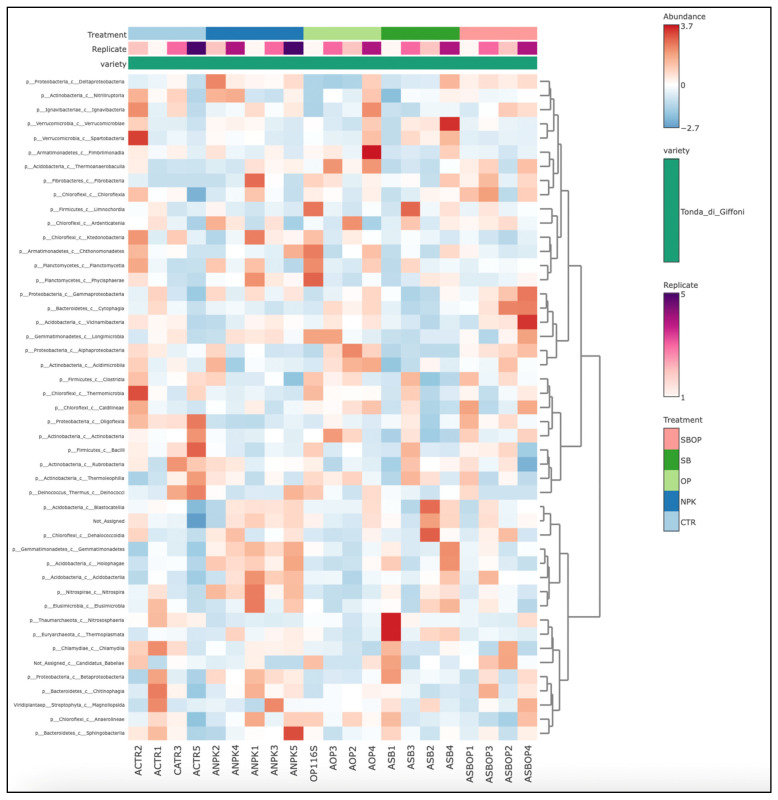
Bacteria heatmap of relative abundance at the class level.

Heatmaps representing bacterial diversity at a class level (Figure 9) indicated that SBOP treatment showed high abundance for *Gammaproteobacteria* and *Bacilli,* suggesting that this treatment created a favorable environment for the proliferation of these classes. In contrast, the SB treatment showed a low abundance of *Alphaproteobacterial* and *Actinobacteria*. Treatment with SB varied between replicates, showing a less uniform response than the other treatments. OP treatment appears less favorable for the proliferation of *Acidobacteria* and *Anaerolineae*, indicating a possible negative impact of OP treatment on this class of bacteria. The NPK treatment showed a variable abundance, especially in the classes *Betaproteobacteria* and *Clostridia*. Soils without treatment (CTR) showed that the abundance of the bacterial classes is generally moderate to low, especially for the classes *Planctomycetacia* and *Verrucomicrobiae*. The dendrogram shows how the bacterial classes are grouped according to their abundance. Classes such as *Gemmaproteobacteria* and *Bacilli* tend to cluster together, indicating a similar response to treatments. In contrast, the upper dendrogram shows how samples with similar abundance patterns cluster together. This clustering confirms that SBOP-treated samples tend to have more similar abundance profiles than other treatments.

**Figure 10:**
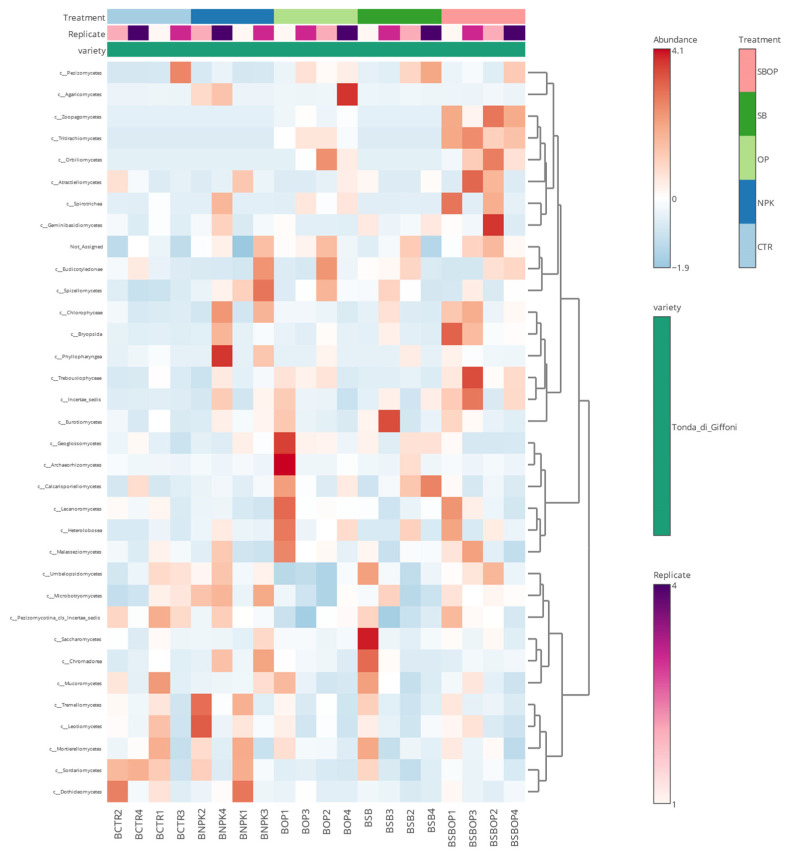
Fungi heatmap of relative abundance at the class level.

A heatmap of fungi at the class level (Figure 10) shows that the SBOP treatment exhibits high abundance for several taxa, and in particular, this treatment could promote the growth of fungi belonging to these groups, suggesting a favorable environment for the proliferation of *Dothideomycetes* and *Eurotiomycetes*. The SB treatment shows a variable abundance pattern, with moderate *Saccharomycetes* and a higher abundance of *Sordariomycetes*. The effect of SB treatment varied between replicates, suggesting a less uniform response than other treatments. OP treatment generally shows low abundance for many taxa, especially in *Geoglossomycetes* and *Leotiomycetes.* The NPK treatment shows considerable variation in the abundance of taxa: a high abundance of *Orbiliomycetes* and a low abundance of *Sordariomycetes*. The CTR treatment shows a generally lower abundance for several taxa: *Agaricomycetes* and *Trebouxiophyceae,* compared to OP and SBOP. The dendrogram shows how taxa are grouped according to their abundance. The taxa *Dothideomycetes Eurotiomycetes* tend to cluster together, indicating a similar response to treatments.

## 4. Discussion

The combined application of chemical and organic fertilizers has previously been documented to offer several advantages over synthetic fertilizers, including a balanced nutrient supply and a reduction in the environmental risks associated with excessive usage [40,41]. While some studies [42,43,44] suggest that short-term effects from applying organic fertilizers may not significantly impact microbial richness due to competition between microbes in bio-organic fertilizers and the existing local bacterial community, others present a contrasting view. For instance, Tian et al. [44] reported a decrease in soil bacterial diversity following organic fertilizer application, whereas other studies [45,46] highlighted the potential of organic fertilizers to enhance the soil microbiome, promoting bacterial growth and increasing population diversity.

Our study demonstrated that the combined fertilizer treatment, composed of organic, composted olive pomace (including a manure component) and sulfur and bentonite minerals, enhanced the microbial abundance in a similar manner to organic fertilizer (OP), rather than with sulfur-bentonite fertilizer (SB) alone.

Our research indicates that adding just 5% of organic material to sulfur-based mineral fertilizer can leverage beneficial increases in various diverse bacteria and fungi, offering a potential improvement in soil quality.

This observation agrees with the study conducted by Marra et al. [47] who demonstrated that bacterial communities were influenced by a sulfur-bentonite-based fertilizer combined with organic components (orange waste) in alkaline soils, at the same application rates used in our study, whilst increases in fungal communities were correlated with an organic fertilizer (horse manure), and actinomycetes were linked to NPK treatments. A recent study by Maffia et al. [48] further showed that compost derived from olive pomace, similar in composition to the one used in this study, significantly increased the number of actinomycete, fungal, and other bacterial taxa compared to the control and other treatments. However, these studies relied on traditional microbial counting methods, which have inherent limitations, such as selective culturing and under-representation of non-culturable microbes. Despite the positive increase in microbial abundance/diversity with 5% organic matter, the optimal fertilization rate at which to achieve maximum increases still requires further experimental verification. For example, Han et al. [49] found that 10–30% organic fertilizer significantly increased bacterial diversity in maize, while others argue for a higher percentage [50].

### 4.1. Effect of Fertilization on Microbial Composition and Biodiversity

At the phylum level, we observed that *Actinobacteria* and *Proteobacteria* were the dominant bacterial groups in the rhizosphere of *Corylus avellana* across all treatments. The increased abundance of *Actinobacteria* in the olive pomace compost (OP) treatment is noteworthy, as this bacterial phylum plays a key role in soil organic matter turnover and the breakdown of complex molecules such as cellulose and polycyclic aromatic hydrocarbons [51,52]. Similarly, the increased presence of *Proteobacteria* in the SBOP and OP treatments may be explained by the rise in soil carbon content following the application of organic amendments, as observed in previous studies [53]. As α-, β-, and γ-*Proteobacteria* are classified as “copiotrophs” that utilize labile carbon for growth, they tend to thrive in nutrient-rich environments [54,55], such as those in the OP and SBOP treatments.

Interestingly, an increase in *Acidobacteria* was also observed across all treatments, particularly in the SB treatment. According to Kalam et al. [56], these bacteria possess genes that enable them to survive in and competitively colonize the rhizosphere, fostering beneficial relationships with plants. Additionally, *Acidobacteria* are equipped with genes that enable them to metabolize both inorganic and organic nitrogen sources, effectively reducing nitrates, nitrites, and nitric oxide [57].

While *Firmicutes* were identified as the fifth most abundant phylum, the genus *Bacillus*—a member of *Firmicutes*—was the most dominant genus across treatments. *Bacillus* is a highly adaptable bacterium capable of surviving adverse environmental conditions by forming spores and degrading organic materials such as cellulose. Though *Firmicutes* as a whole may be less abundant relative to other phyla, the dominance of *Bacillus* is consistent with its ability to thrive in diverse soil environments [58]. However, while several studies have shown that *Bacillus* abundance increases with the application of organic fertilizers [59,60], our results align with Wu J et al. [61], who observed a sharp decline in *Bacillus* relative abundance in fertilized soils compared to unfertilized soils.

Regarding fungi, *Ascomycota* and *Streptophyta* were the dominant phyla in the rhizosphere of *Corylus avellana* across all treatments. Our study showed a lower relative abundance of *Ascomycota* in most treatments compared to the control (CTR). This finding is in line with a recent study by Sivojienė et al. [62], which also noted a reduction in the abundance of *Ascomycota* following organic fertilizer application, such as poultry manure. This could have been attributed to the more efficient competition for resources by other microorganisms or their better adaptation to new soil chemical conditions, such as fungi from the genus *Talaromyces*. This genus was particularly abundant in the OP treatment. A similar study reported a negative correlation between *Basidiomycota* abundance and high levels of total nitrogen (TN), soil organic carbon (SOC), and soil moisture (SM), but a positive correlation with aromatic substance availability (SA) [63]. This suggests that *Basidiomycota* may thrive in soils with high carbon and nitrogen content but where aromatic substances are more prevalent, as is often the case with organic fertilizers.

The second most abundant fungal phylum was *Streptophyta*, a group of land plants and algae that may compete with crops for nitrogen [64,65,66], potentially coming from contamination of the water used for this field experiment. Considering the competitive role of *Streptophyta* in nutrient uptake, particularly nitrogen, the SB treatment was observed to be associated with the smallest increase in *Streptophyta* abundance (25%) so could, therefore, be considered the most favorable treatment for minimizing competition between crops and this phylum.

Fungi belonging to the genus *Talaromyces* were notable for their role as primary decomposers of plant residues and as antagonists towards other fungi [67]. Some species of *Talaromyces* secrete organic acids and phosphatase, aiding in the dissolution of inorganic calcium phosphate and phosphate ester, which promotes phosphorus uptake by plants [68,69]. In our study, the SB treatment demonstrated the highest response in *Talaromyces* abundance compared to the control. This may have been due to the soil acidification induced by sulfur, which creates a favorable environment for *Talaromyces*; however, additional assessments of soil pH would be required to verify this. Additionally, bentonite present in SB treatments could have contributed to improvements in soil structure, moisture retention, and increases in phosphorus availability, thereby enhancing the growth-promoting effects of *Talaromyces* on plants.

The increase in alpha-diversity, particularly bacterial species richness as measured by the Chao1 index, in soils treated with sulfur bentonite (SB) and organic sulfur bentonite (SBOP), is consistent with the findings of Damo et al. [70], who reported that sulfur applications significantly increased both microbial abundance and diversity compared to sulfur-free treatments. Notably, when comparing SB and SBOP, the organic component of compost in the SBOP treatment plays a critical role in further enhancing species richness for both bacteria and fungi, even in short-term treatments. This observation aligns with other studies [71,72], which emphasize the pivotal role of organic matter in fostering microbial biodiversity.

Alpha diversity, as quantified through the Chao1 index, reflects the richness or the number of distinct species in the soil microbial community. Our results show that the inclusion of even a small percentage (5%) of organic material in the SBOP treatment resulted in a significant increase in fungal richness compared to the control (CTR), underscoring the importance of organic components in soil biodiversity. These results corroborate the findings of Hu et al. [43], who demonstrated that the use of organic fertilizers can significantly enhance microbial composition even after short-term application. This suggests that the composition and quality of organic matter are key factors in promoting soil alpha-diversity, a conclusion also supported by Guo et al. [73].

In terms of beta-diversity, which refers to the differences in community composition between treatments, the Bray–Curtis dissimilarity index highlighted the strong influence of fertilizer type on microbial community structure. Organic amendments, such as compost (OP) or sulfur bentonite, supported the establishment of distinct microbial communities compared to mineral fertilizers like NPK. This differentiation suggests that the organic matter, in combination with other components like sulfur, significantly reshapes soil microbial populations, which is a phenomenon widely observed in soil ecology [43]. However, the high variability within treatment groups, as if often seen in complex soil environments, may obscure clear clustering patterns, which can be influenced by several factors, including nutrient availability, microbial interactions, and soil structure.

This variability in microbial response suggests a nuanced interaction between the fertilizer types and the inherent soil microbial community, where the specific components of the organic matter (e.g., compost) play a critical role in driving both alpha and beta-diversity changes.

### 4.2. Sulfur with Organic Matter Enhances Beneficial Microbial Component

The results obtained with LEfSe (linear discriminant analysis effect size) analysis showed significant differences in bacterial and fungal communities between different fertilization treatments, allowing us to identify key taxa that influence soil health and plant-soil interactions. In particular, fertilizers containing organic components, such as the OP and SBOP, favored the proliferation of bacteria and fungi closely linked to the nutrient cycle, improving nutritional efficiency and soil quality.

Among bacteria, *Thiobacillus* was found to have a significantly higher relative abundance in SBOP-treated soils, emphasizing the critical role of this genus in the sulfur cycle. *Thiobacillus* oxidises the elemental sulfur and reduced compounds such as H₂S and thiosulphates, converting them to sulphate (SO_4_^2−^), a process that not only enriches the soil with essential sulphates, but also facilitates the solubilization of other nutrients, including phosphates and micronutrient metals [74]. This microbial activity promoted by the organic component of sulfur fertilizer highlights how the incorporation of organic materials can enhance the bioavailability of key nutrients.

*Pseudoxanthomonas,* another bacterial genus found with significant abundance in OP and SBOP treatments, contributes to the decomposition of complex organic compounds, such as lignin and cellulose, improving nutrient availability to plants. The presence of this genus has also been associated with an increase in soil biodiversity, with potential positive effects on rhizosphere health [75,76]. Along with bacteria such as *Pseudoxanthomonas*, fungi such as *Thermomyces* were also found to be more abundant in SBOP treatments. *Thermomyces* are crucial for the degradation of lignocellulose and facilitate the mineralization of organic matter, supporting the carbon and nitrogen cycle in the soil [77,78]. This synergy between bacteria and fungi creates a nutrient-rich environment that supports plant growth and health.

Bacteria belonging to the genus *Reyranella*, present in SBOP and OP treatments, play a significant role in carbon transformation and stabilization of soil microbial communities, contributing to soil resilience and health. This genus is known for its ability to adapt to variable soil conditions and interact positively with other microorganisms, improving the stability of microbial communities and promoting effective biogeochemical cycles. In parallel, the thermophilic fungus *Pseudallescheria* has been found in abundance with the addition of organic fertilizers, especially with SBOP, playing an important role in mineralizing organic matter and improving soil fertility [79,80]. This ability to decompose organic matter makes *Pseudallescheria* a key player in the nutrient cycle, particularly in agricultural systems using organic fertilizers.

The genus *Phaselicystis* was also present in abundance in the OP and SBOP treatments, and is a crucial component linking soil nutrient cycling and plant defense. This genus, and in particular the only known species *Phaselicystis flava*, contributes to the degradation of complex organic compounds, improving the availability of nutrients such as organic carbon and nitrogen, which are essential for plant growth support [81]. However, *Phaselicystis* also stands out for its role in plant defense due to its ability to produce arachidonic acid, an allelopathic metabolite that mediates interactions between microorganisms in the soil, particularly between arbuscular mycorrhizal fungi and bacteria [82]. Arachidonic acid not only modulates microbial interactions, but also recruits beneficial microorganisms into the rhizosphere, promoting nutrient turnover and protecting plants from abiotic and biotic stresses [82,83].

Next to bacteria, many fungi also play a crucial role in plant defense. *Tritirachium,* a fungus abundant in OP and SBOP treatments, is known for its entomopathogenic properties and protease production, which contribute to plant protection from pest attacks [84]. This highlights how the organic component of fertilizers, in addition to improving nutrition, promotes the growth of microorganisms with defensive properties.

*Dactylella*, another fungus present exclusively in OP and SBOP treatments, is known for its ability to trap nematodes and for its use in the biocontrol of fungal pathogens, contributing to a synergistic approach in natural plant protection. Its presence suggests a reduction in the need for external chemical inputs, improving soil resilience and promoting more sustainable agriculture [85]. Finally, the fungus *Chaetomium*, known to produce numerous metabolites with antifungal and photoprotective activity, showed a decrease in all treatments compared to the control, especially with OP. This could indicate that enriching the soil with treatments that promote beneficial microorganisms reduces the need for the protective action *Chaetomium* provides in untreated soil, highlighting the complexity of microbial interactions in an organic fertilization context [86,87].

## 5. Conclusions

The present study demonstrates that the combined application of chemical and organic fertilizers—specifically, an innovative blend of composted olive pomace and mineral constituents such as sulfur and bentonite—represents a promising strategy for enhancing soil biodiversity. Our findings indicate that even a modest addition of organic matter (5%) to sulfur-based fertilizers can significantly enhance both bacterial and fungal diversity, yielding effects comparable to those observed with purely organic fertilizers. These results underscore the potential of mixed fertilization strategies to sustain soil health and promote plant growth by optimizing microbial diversity and functionality. Notably, we observed substantial changes in the rhizosphere microbiome of *Corylus avellana* following the application of various fertilizers, which influenced microbiome composition. The application of organic fertilizers, including composted olive pomace and a sulfur-bentonite-olive pomace combination, notably improved the Chao1 index for bacterial and fungal communities. For example, the SBOP treatment showed an increase in Chao1 index compared to the control of 18% for bacterial communities and 22% for fungal communities compared to the unfertilized soil (CTR). The OP treatment, on the other hand, showed an increase of 16% for bacteria and 20% for fungi compared to the unfertilized soil (CTR). It is crucial to emphasize that our assessments focus on the short-term effects of these fertilization strategies. Future research will continue to explore the long-term implications of these practices on soil health and sustainability. Nonetheless, the current findings are promising, indicating positive outcomes for agricultural sustainability and the effective use of fertilizers, thereby contributing to a more balanced and sustainable approach to soil management.

## Figures and Tables

**Table 1:** Chemical properties of composted olive pomace. The data are the mean of three replicates ± standard deviation.

Chemical Properties	Value
pH (H_2_O)	7.8 ± 0.04
EC (mS cm^−1^)	2.6 ± 0.27
Moisture (g kg^−1^ fw)	383 ± 0.3
C% (g kg^−1^ dw)	426 ± 0.4
Total N (g kg^−1^ dw)	19.6 ± 0.1
C/N	21.7 ± 0.3
Na^+^ (mg g^−1^ dw)	4.35 ± 0.02
NH4^+^ (mg g^−1^ dw)	1.50 ± 0.01
K^+^ (mg g^−1^ dw)	9.13 ± 0.06
Mg^2+^ (mg g^−1^ dw)	1.45 ± 0.43
Ca^2+^ (mg g^−1^ dw)	14.06 ± 1.4
Cl^−^ (mg g^−1^ dw)	0.02 ± 0.5
PO4^3−^ (mg g^−1^ dw)	0.40 ± 0.4
SO4^2−^ (mg g^−1^ dw)	0.18 ± 2.9
WSB (mg TAE g^−1^ dw)	2.5 ± 0.05

**Table 2:** Composition of different fertilizers.

Fertilizers	Composition
Sulfur Bentonite + Olive Pomace (SBOP)	5% of composted olive pomace recovered using a two-phase oil mill 10% of bentonite clay 85% of elemental sulfur.
Sulfur Bentonite (SB):	90% of elemental sulfur 10% of bentonite clay.
Composted Olive Pomace (OP)	34% of composted olive pomace recovered using a two-phase oil mill 33% of buffalo manure 33% of a mixture consisting of wood defibrate and olive leaves.
Synthetic fertilizer (NPK)	20% of N 10% of P_2_O_5_ 10% of K_2_O

## Data Availability

All sequences were deposited at the European Nucleotide Archive (ENA, http://www.ebi.ac.uk/ena accessed on 15 January 2024) under project number PRJEB70816 for bacteria and PRJEB68325 for fungi.

## Supplementary Materials

The following supporting information can be downloaded at: https://www.mdpi.com/article/10.3390/life14121633/s1, Table S1: Bacteria Beta diversity Bray–Curtis. The table below summarizes the result of pairwise PERMANOVA analysis. The multi-testing adjustment is based on Benjamini–Hochberg procedure (FDR); Table S2: Fungi Beta diversity Bray–Curtis. The table below summarizes the result of pairwise PERMANOVA analysis. The multi-testing adjustment is based on Benjamini–Hochberg procedure (FDR); Table S3: Bacteria LEfSe results with a *p* value cut of 0.05 and Log LDA score of 2.0. The table below shows the 44 features ranked by their *p* values that are statistically significant; Table S4: Fungi LEfSe results with a *p* value cut of 0.05 and a Log LDA score of 2.0. The table below shows the 44 features ranked by their *p* values that are statistically significant.
