# Supplementary material for: Transforming Agricultural and Sulfur Waste into Fertilizer: Assessing the Short-Term Effects on Microbial Biodiversity via a Metagenomic Approach
Source: Life (Basel). 2024 Dec 9;14(12):1633. doi: 10.3390/life14121633 (PMC11677321; doi:10.3390/life14121633)
Supplement: Supplementary file 1 [file life-14-01633-s001.zip › life-3332136-supplementary.pdf]

Table S1. Bacteria Beta diversity Bray-Curtis. The table below summarizes the result of pairwise PERMANOVA analysis. The multi-testing adjustment is based on Benjamini-Hochberg procedure procedure (FDR).

Pair	F-value	R-squared	P-value	FDR
CTR vs NPK	2.5627	0.26799	0.037	0.23
OP vs NPK	2.5105	0.26398	0.046	0.23
SB vs OP	1.9005	0.24055	0.079	0.2375
SBOP vs NPK	1.8262	0.2069	0.095	0.2375
SB vs SBOP	1.5699	0.20739	0.158	0.316
SB vs CTR	1.6253	0.21314	0.201	0.335
SB vs NPK	0.94462	0.1189	0.497	0.625
SBOP vs CTR	0.8891	0.12906	0.5	0.625
OP vs CTR	0.84228	0.1231	0.625	0.69444
OP vs SBOP	0.67989	0.10178	0.775	0.775

Table S2. Fungi Beta diversity Bray-Curtis. The table below summarizes the result of pairwise PERMANOVA analysis. The multi-testing adjustment is based on Benjamini-Hochberg procedure procedure (FDR).

Pair	F-value	R-squared	P-value	FDR
OP vs NPK	1.5973	0.18579	0.032	0.19667
OP vs CTR	2.3094	0.27793	0.047	0.19667
SBOP vs CTR	2.4954	0.29374	0.059	0.19667
SBOP vs NPK	1.5329	0.17964	0.08	0.2
SB vs CTR	1.2429	0.1716	0.329	0.56286
SB vs SBOP	1.1473	0.16053	0.356	0.56286
CTR vs NPK	1.0715	0.13275	0.394	0.56286
SB vs NPK	0.99898	0.12489	0.538	0.6725
SBOP vs OP	0.61709	0.093257	0.787	0.87444
SB vs OP	0.55653	0.084882	0.94	0.94

Table S3 Bacteria LEfSe results with P value cut of 0.05 and Log LDA score 2.0. The table below shows the most 44 features ranked by their p values that are statistically significant.

	Pvalues	FDR	CTR	NPK	OP	SB	SBOP	LDAscore
p__Candidatus_Rokubacteria	0.0079255	0.39859	47363	93573	36597	94259	66045	4.46
o__Oceanospirillales	0.0085641	0.39859	167.08	237.66	914.45	319.95	788.61	2.57
g__Pseudonocardia	0.011346	0.39859	53544	40963	68022	38007	58464	4.18
p__Armatimonadetes	0.011604	0.39859	78267	160810	87023	146980	104390	4.62
f__Bryobacteraceae	0.011712	0.39859	251.4	315.12	1176.3	43.415	1180	2.76
f__Micromonosporaceae	0.014241	0.39859	103270	74351	138160	69523	147850	4.59
f__Steroidobacteraceae	0.014522	0.39859	12551	14513	19760	11867	25954	3.85
o__Sphingomonadales	0.017217	0.39859	5047.6	9244.3	9960.7	4163	9617	3.46
f__Rhodobacteraceae	0.018303	0.39859	4329.2	2934.7	11488	3183.3	16851	3.84
f__Hyphomonadaceae	0.020315	0.39859	1699.6	2001.3	4988	1438.7	4690.4	3.25
o__Rhizobiales	0.020656	0.39859	281270	251420	341210	197400	318470	4.86
g__Phaselicystis	0.020886	0.39859	3608.9	2186.8	4187.6	3069	5281	3.19
g__Thiobacillus*	0.021653	0.39859	0	0	132.31	980.01	2639.8	3.12
f__Nitrososphaeraceae	0.022037	0.39859	365770	136510	195610	412810	219290	5.14
g__Hirschia	0.022629	0.39859	1824.4	1832.7	3201.9	655.7	4738.3	3.31
f__Iamiaceae	0.023782	0.39859	1142.5	1663.3	3328.4	820.35	2807.1	3.1

**g__Pseudoxanthomonas**	0.024931	0.39859	86.696	0	1772.3	0	2182.3	3.04
**f__Geminicoccaceae**	0.026433	0.39859	30525	23287	40540	21616	35829	3.98
**c__Nitrospira**	0.028002	0.39859	61392	112270	46550	73893	62666	4.52
**o__Xanthomonadales**	0.028063	0.39859	2111.8	4958.5	3442.9	1607.7	2903.8	3.22
**f__Sandaracinaceae**	0.028685	0.39859	12045	5757	8704.8	4296.4	17704	3.83
**f__Burkholderiaceae**	0.029953	0.39859	14717	24321	10934	21679	14969	3.83
**g__Paludibaculum**	0.032001	0.39859	297.41	768.17	2496	815.61	2486.5	3.04
**c__Thermoplasmata**	0.032353	0.39859	7879	4832	2444	16925	6296.4	3.86
**f__Gemmatimonadaceae**	0.038068	0.39859	366070	661960	449430	572720	480250	5.17
**o__Bryobacterales**	0.038525	0.39859	809.31	1822.4	1963	955.91	2069.5	2.8
**f__Thermoanaerobaculaceae**	0.040188	0.39859	3607	4833.9	7158.7	3349.2	5768.9	3.28
**f__Hyphomicrobiaceae**	0.04337	0.39859	26456	23622	33992	17141	37036	4
**f__Methylophilaceae**	0.043923	0.39859	172.63	1387.2	409.45	294.5	673.63	2.78
**c__Deltaproteobacteria**	0.04482	0.39859	34735	63729	39249	48048	48905	4.16
**f__Rhodospirillaceae**	0.045501	0.39859	19937	16884	25395	13608	22938	3.77
**g__Nitrosospira**	0.045857	0.39859	84.064	1536.7	207.67	246.87	301.51	2.86
**f__Xanthobacteraceae**	0.046043	0.39859	118000	92957	122970	84835	121650	4.28
**o__Micrococcales**	0.046043	0.39859	21871	17483	20204	7061.7	27174	4
**g__Anaeromyxobacter**	0.046292	0.39859	22736	27057	11164	23241	20608	3.9
**g__Adhaeribacter**	0.047013	0.39859	1527.3	366.16	1566.5	162.49	1353	2.85
**f__Cellulomonadaceae**	0.047757	0.39859	930.45	355.28	1460.3	344.32	1853.4	2.88
**o__Cytophagales**	0.049375	0.39859	1950.3	2206.4	3219.1	767.97	3934.9	3.2
**f__Ruminococcaceae**	0.049452	0.39859	506.23	33.101	292.27	344.03	849.23	2.61
**c__Nitrososphaeria**	0.049727	0.39859	2085.1	1252.2	170.03	4820.5	2492.4	3.37
**o__Gemmatimonadales**	0.049774	0.39859	26693	60167	37011	38090	34892	4.22

Table S4. Fungi LEfSe results with P value cut of 0.05 and Log LDA score 2.0. The table below shows the most 28 features ranked by their p values that are statistically significant.

	**Pvalues**	**FDR**	**CTR**	**NPK**	**OP**	**SB**	**SBOP**	**LDAscore**
**s__Tritirachium_oryzae**	0.00058283	0.062815	0	0	1175.1	0	3049.5	3.18
**s__Zoopagales_sp**	0.00058283	0.062815	0	0	932.99	0	8488.6	3.63
**o__Zoopagales**	0.00065432	0.062815	0	0	529.14	0	1401.6	2.85
**s__Podospora_pyriformis**	0.0014181	0.07733	78.761	395.95	32137	0	178890	4.95
**s__Pleurotheciella_sp**	0.0015757	0.07733	0	98.987	1897.6	0	14383	3.86
**s__Monascus_pallens**	0.001611	0.07733	0	230.97	1784.6	0	5397.2	3.43
**s__Zopfiella_sp**	0.0019677	0.07758	0	68.043	1135.5	0	2478.7	3.09
**g__Thermomyces**	0.002155	0.07758	0	958.93	37246	35.385	105080	4.72
**s__Dactylella_cylindrospora**	0.0028479	0.084881	0	0	2097.7	0	3055.2	3.18
**s__Ophiostomatales_sp**	0.0029473	0.084881	0	131.98	526.09	0	2847.3	3.15
**f__Ophiocordycipitaceae**	0.0047992	0.12255	2687.7	6387.9	28270	3672.5	10734	4.11
**g__Pseudallescheria**	0.0051061	0.12255	0	32.996	1445	38.286	4732	3.37
**o__Tubeufiales**	0.0055394	0.12272	0	230.97	16084	0	12939	3.91
**c__Agaricomycetes**	0.0060289	0.12402	435.66	318.87	5502.4	332.97	20390	4
**p__Rozellomycota**	0.0070998	0.1338	398.99	55.16	1429	79.215	2547.9	3.1
**f__Microascaceae**	0.0074335	0.1338	160.5	1001.6	4303.2	645.4	9720.8	3.68
**g__Chaetomium**	0.013263	0.21488	91224	73520	27767	47879	47176	4.5
**s__Veronaea_botryosa**	0.01343	0.21488	236.28	32.996	441.78	0	1109	2.74
**p__Ascomycota**	0.015144	0.22955	73965	80363	307470	60175	571300	5.41
**s__Zopfiella_attenuata**	0.018293	0.26293	28645	12007	450.71	9003.1	6358.7	4.15
**s__Tremellomycetes_sp**	0.019172	0.26293	159.95	164.98	10412	368.68	8447.8	3.71
**g__Podospora**	0.023847	0.3116	587.38	697.56	2376.4	989.77	16109	3.89
**g__Peziza**	0.024885	0.3116	0	230.44	4761.3	176520	1322	4.95
**g__Malbranchea**	0.027499	0.32227	553.65	0	945.45	2271.4	493.76	3.06
**f__Pezizaceae**	0.027975	0.32227	1950.1	1248.1	8839.5	16211	16582	3.88
**s__Spizellomycetales_sp**	0.043144	0.4779	117.31	680.73	565.7	181.9	650.51	2.45
**s__Ciliophora_sp_1**	0.048326	0.50216	27040	16503	6251.1	6164.2	20437	4.02
